# Payload for Contact Inspection Tasks with UAV Systems

**DOI:** 10.3390/s19173752

**Published:** 2019-08-30

**Authors:** L. M. González-deSantos, J. Martínez-Sánchez, H. González-Jorge, M. Ribeiro, J. B. de Sousa, P. Arias

**Affiliations:** 1 Applied Geotechnologies Group, Dept. Natural Resources and Environmental Engineering, University of Vigo, Campus Lagoas-Marcosende, CP 36310 Vigo, Spain; 2 Applied Geotechnologies Group, Dept. Natural Resources and Environmental Engineering, School of Aerospace Engineering, University of Vigo, Campus Lagoas, CP 32004 Ourense, Spain; 3 Underwater Systems and Technology Laboratory, Dept. of Electrical and Computer Engineering, School of Engineering (FEUP), University of Porto, 4200-465 Porto, Portugal

**Keywords:** autonomous navigation, contact inspection, NDT, UAV, payload

## Abstract

This paper presents a payload designed to perform semi-autonomous contact inspection tasks without any type of positioning system external to the UAV, such as a global navigation satellite system (GNSS) or motion capture system, making possible inspection in challenging GNSS- denied sites. This payload includes two LiDAR sensors which measure the distance between the UAV and the target structure and their inner orientation angle. The system uses this information to control the approaching of the UAV to the structure and the contact between both, actuating over the pitch and yaw signals. This control is performed using a hybrid automaton with different states that represent all the possible UAV status during the inspection tasks. It uses different control strategies in each state. An ultrasonic gauge has been used as the inspection sensor of the payload to measure the thickness of a metallic sheet. The sensor requires a stable contact in order to collect reliable measurements. Several tests have been performed on the system, reaching accurate results which show it is able to maintain a stable contact with the target structure.

## 1. Introduction 

Monitoring the state of a structure is essential for structural maintenance and failure prevention [1]. In some cases, these inspection tasks have to be carried out in difficult to access structures, such as wind farms [2], bridges [3,4,5,6] or dams [7]. In addition, these require a significant investment, so they need to run properly for the project to be profitable [8].

The unmanned aerial vehicles (UAV) market has grown during the past years and it will continue growing in the coming ones [9] showing applicability in many fields such as surveying, video production and, being of the utmost importance, industrial inspection. The main advantage of using UAVs for inspection tasks is their ability to access complex and dangerous areas.

Within this context and in most of the cases, the sensors used as payloads for UAV-based inspection are cameras or Light Detection and Ranging (LiDAR), that support the remote documentation of the target structure. RGB cameras are used to detect damaged areas in joints of bridges by taking some aerial images, which are then analyzed to look for possible structural damages [10]. Araar et al. presented an autonomous power lines inspection method [11], where an UAV equipped with a RGB camera achieves real time tracking. Omari et al. presented an industrial inspection technique using UAVs [12], where a visual inertial stereo camera is used for localization as well as to generate a dense 3D map for obstacle avoidance in real time. Mader et al. presented a UAV-based building inspection method [13], that combines a LiDAR sensor with a thermal and multispectral camera for the inspection of different structures such as bridges or dams. All these multisource data can be integrated in a monitoring system, providing a powerful tool for levee monitoring and evaluation [14]. Other UAV systems are equipped with a Ground Penetrating Radar (GPR) sensor that allows the system to detect metallic and dielectric targets that are underground [15].

While UAV-based structure inspection is mostly performed with close range remote sensing payloads, data collection for structural monitoring is often based on Non Destructive Testing (NDT) contact sensors [16]. Hence, there is an increasing interest in integrating both UAV and NDT contact monitoring sensors and there are several examples on this topic in the literature. Trujillo et al. presented a UAV equipped with a six Degrees of Freedom (DoF) manipulator installed in the UAV that makes contact with the structure [17]. This system uses the joint’s sensors of the robotic arm for the position control of the UAV, but the approximation to the structure is done manually by the pilot. Sanchez-Cuevas et al. presented a system that is able to perform contact inspection tasks on the bottom part of a bridge [18]. In this case, the ceiling effect is used to maintain the contact, and as in the previous case, the UAV has to be manually piloted to make the contact. Watanabe et al. have developed a similar UAV system, but adding a rotational movement to the motors, which allows the system to climb vertical walls [19]. Hamaza et al. developed a manipulator with two DoF installed in a quadcopter [20], which uses a Vicon motion capture system for the position control. Zhang et al. have developed a UAV system similar to the previous one, but with just one DoF on a linear joint, that has a spring [21]. Albers et al. developed a UAV system with a horizontal motor that pushes the system against the wall on which the contact has to be done [22].

The flight control algorithms to make the UAV capable of being in stable contact with the structure are complex. Different control strategies have been developed for this purpose. In some cases common control algorithms are used, such as PD-PID algorithms [23]. In other cases, more complex algorithms are used, determining the dynamic model of the system to control it [24], or with a Cartesian Impedance Control method [25]. 

All these works use external positioning systems that may be restricted or even unavailable in real cases, such as inspections in wind turbines or bridges, since they need some external components to be installed in the area where the contact inspection will be done. Motion capture systems based on cameras cannot be used in these cases because this implies that a camera system has to be installed and calibrated in the area where the inspection will be done. Simultaneous Localization and Mapping (SLAM) algorithms are one of the positioning systems most used for this purpose, but if cameras are used, the images need to have some ‘texture’, which means that it does not work well for example in the case of wind turbines. In this case the structure is smooth and completely white and does not provide the required texture. Kang et al. used an Ultrasonic Beacon System for UAV positioning [26], but as in the case of the motion capture systems, to work with this system a series of beacons have to be installed and calibrated. Global Positioning System (GPS) reliability may also be restricted, since the GPS signal is usually poorer (or even denied) in the neighborhood of large structures, which makes it unusable to control the contact.

In this paper, a smart payload for contact inspection tasks is presented. This payload has a control system based on two LiDAR sensors that are used to calculate the distance and the angle the UAV forms regarding to a structure. This information is used to control the UAV to make a slow approach to the structure, ending with a stable contact. An ultrasonic sensor to measure the thickness of metal sheets is used as inspection tool, but the system is designed to use any kind of sensor that needs contact. The main innovation of this payload is that it is a complete system that makes the UAV able to real time control the position regarding a structure and it does not require complex algorithms to work. This system is not completely autonomous, since it needs an operator to work. The main objective is to give the operator all the tools needed to be able to carry out contact inspection tasks with a UAV. 

The manuscript is organized as follows: Section 2 describes the payload and control algorithms. Section 3 reports the results obtained from applying the payload to a study that simulate a real study case a discussion of those results. Finally, Section 4 concludes this work.

## 2. Methodology

**Figure 1:**
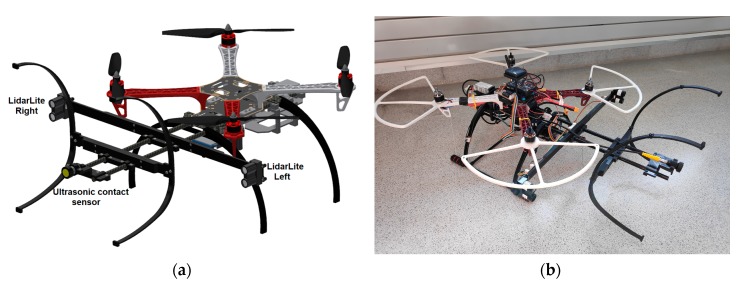
(**a**) Simplified CAD design of the UAV with the payload. (**b**) Payload installed in the UAV.

The payload has been designed to install it in a F450 quadcopter (DJI, Shenzhen, China) but it can be adapted to any other frames or configurations. The payload is independent of the flight controller. In this case, a Pixhawk open hardware flight controller has been used, with PX4 open software. The payload is composed of two LiDAR distance sensors, a payload controller and all the mechanical components needed to support the sensors (Figure 1). All these mechanical components have been made by 3D printing.

**Figure 2:**
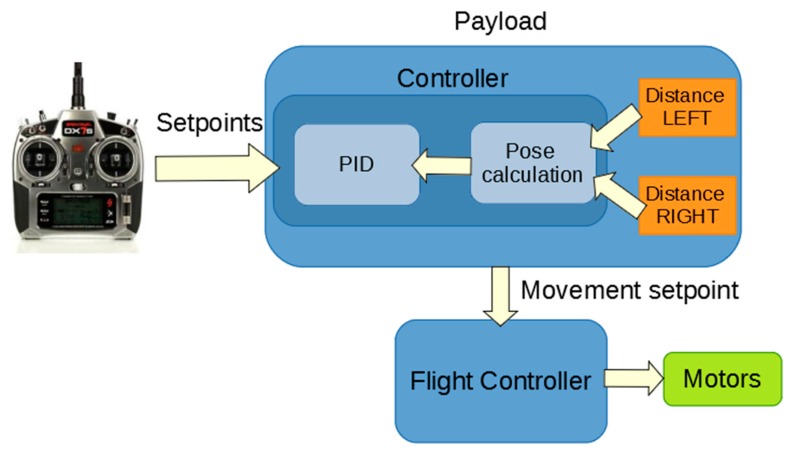
Control diagram.

Figure 2 shows the control diagram of the system. As can be seen, the radio signal is an input for the payload. With the setpoint of the radio signal and the distance from each side of the payload, the system is able to control the approaching to the structure to achieve a stable contact, as will be explained in the following sections. 

**Figure 3:**
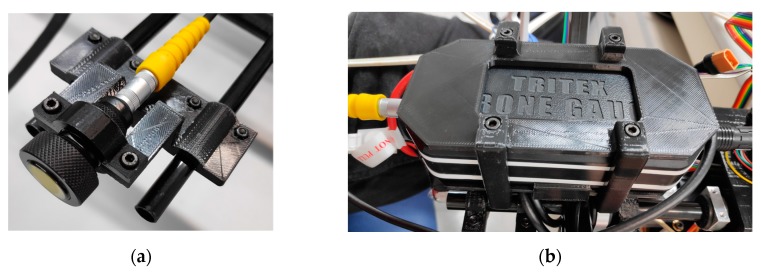
Components of the gauge. (**a**) Probe. (**b**) Gauge body.

The contact sensor used in this case is an ultrasonic sensor from Tritex NDT [27] for the measurement of the thickness of a metal sheet. This sensor has been specifically designed to be mounted onto a UAV. It is composed of a probe, that is the sensitive part of the gauge that have to be in contact with the structure to make the measurement, and the gauge body, that have all the electronic components for process the signal from the probe and communicate the measurement to the PC. This gauge transmits the measurements wirelessly to a PC up to 500 m. A series of supports have been designed in order to install these components in the UAV frame (Figure 3). 

### 2.1. Distance/Angle Measurement System

The distance and the angle of the UAV with regards to the structure are measured using only two LiDAR distance sensors. A previous design has been reported [28], using triangulation distance sensors. In that case, three different sensors are used to achieve a range from 4 cm to 5 m, due to the short range of each sensor used. In that previous work, the system was able to control the distance and the angle of the UAV regarding to the structure, but it was only a distance control, it was not designed to make a contact. 

**Figure 4:**
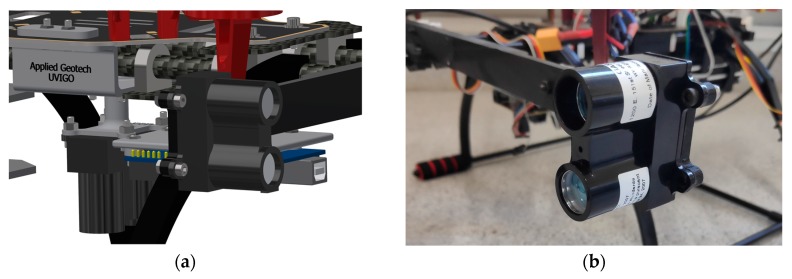
LiDAR sensor used in the designed payload. (**a**) CAD design. (**b**) System photograph.

For the payload presented in the current work, LiDAR Lite V3 distance sensors [29] have been used (Figure 4). These sensors have a resolution of 1 cm, a range up to 40 m and an accuracy of 2.5 cm for distances lower than 5 m.

**Figure 5:**
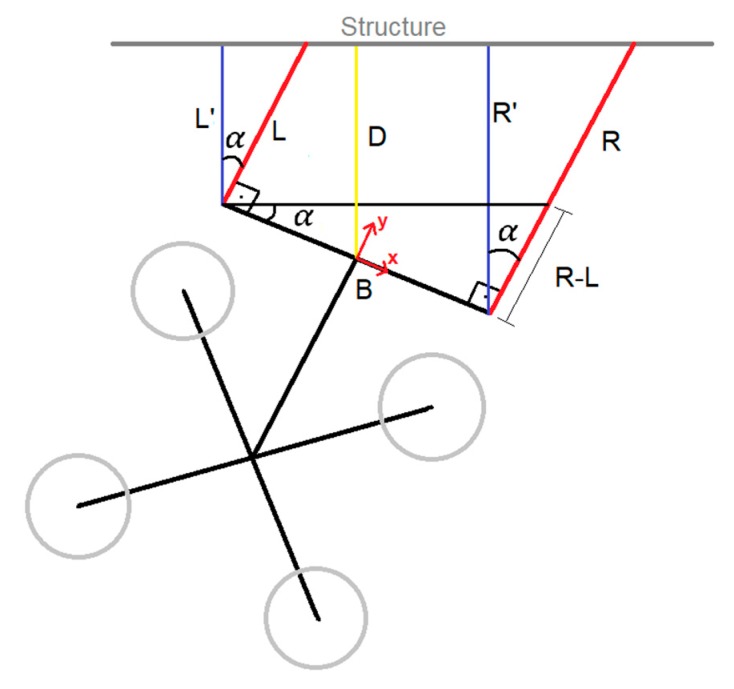
Trigonometric relationship between the measured distances and the UAV pose relative to the structure.

With these two distances measured and the base-line distance between the sensors, the system is able to calculate the distance and the angle formed between the UAV and the structure (Figure 5). Equation (1) is used to calculate the angle formed between the UAV and the structure (α), using the distance measured by the right sensor (R), the distance measured by the left sensor (L) and the base line (B). For the current design, the length of base line is 50.1 cm:

(1) $$\tan\left(\alpha \right)=\frac{R-L}{B}$$

The origin of coordinates system of the payload has been placed in the center of the piece that joints the distance sensors. Once the angle α is obtained, the distance of each side perpendicular to the structure is calculated ($L^{\prime }$ and $R^{\prime }$). Therefore, the distance of the UAV relative to the structure (D) is calculated perpendicular to the structure (Equation (2)):

(2) $$\begin{array}{c} L^{\prime }=L*\cos\left(\alpha \right)\\ R^{\prime }=R*\cos\left(\alpha \right)\\ D=L^{\prime }+\frac{R^{\prime }-L^{\prime }}{2} \end{array}$$

#### Calibration

Once the payload was mounted, it was observed that the LiDAR sensors measured different values for the same distance, despite being the same sensor model. This difference in the measured distance made it necessary to calibrate each sensor separately. In order to do this calibration, a Leica Disto plus laser distance sensor (Leica, Wetzlar, Germany) was used, which has an accuracy of 1 mm. These sensors were calibrated installed in the payload, in this way the offset between the origin of coordinates of each sensor and the origin of coordinates of the payload is within the calibration parameters. The UAV with the payload installed was fixed centered to a calibration support, specifically designed for the calibration. This support has 1.5 m wide and a piece where the laser distance sensor can be placed leveled in the same vertical plane than the origin of coordinates of the payload. The calibration support with the UAV was placed in front of a flat wall, with the sensors pointing at the wall. The system was moved from 35 cm away from the wall, where the sensor was in contact with the wall, up to 2.1 m, always parallel to the wall.

**Figure 6:**
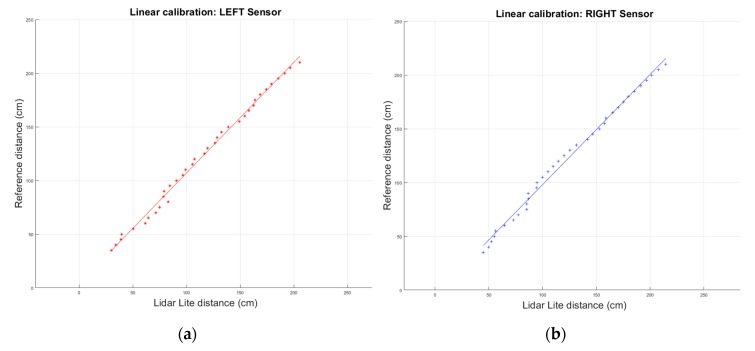
Distance sensor calibration. (**a**) Left sensor. (**b**) Right sensor.

A distance measurement was taken with each sensor every 5 cm, which was then compared with the distance measured by the distance sensor used for the calibration. In this case, the data input in the system is the distance measured by the LiDAR Lite sensors from each side. The data of this comparison can be seen in Figure 6. As can be observed, the sensors behave linearly, as expected for Time of Flight (ToF) LiDAR distance sensors. 

To achieve a fast and accurate calibration function for each sensor, a least-squares fitting algorithm is used to the linear fitting of the data, where x is the distance measured by each Lidar Lite sensor and y is the real distance calibrated. The parameters and the coefficient of determination ($R^{2}$) obtained are showed in Equation (3).

(3) $$\begin{array}{c} Left:\;y=1.0312x+3.9016\;R^{2}=0.994\\ Right:\;y=1.0259x-4.6038\;R^{2}=0.992 \end{array}$$

The value of the coefficient of determination means that the calculated calibration curves fit the sensor model and validates de used approach.

The used LiDAR distance sensors have a resolution of 1 cm, which makes the system unreliable for measuring angles near 0°. Being the baseline distance 50.1 cm, the minimum reliable angle that the system is able to measure (angle resolution) is 2.54° (Equation(4)). In practice, the threshold to ensure reliable measurements was established at ±4°:

(4) $$\alpha_{Min}=\mathrm{atan}\left(\frac{mR-mL}{Baseline}\right)=\mathrm{atan}\left(\frac{2\;\mathrm{cm}}{50.1\;\mathrm{cm}}\right)=2.54^{{}^{\circ}}$$

### 2.2. UAV Control

As aforementioned, one of the main objectives during the design of this payload was to make it independent of the flight controller and adaptable to other frames and configurations. The height control of the UAV is accomplished by the flight controller using a LiDAR sensor in the bottom frame of the UAV, so the payload just sends the height setpoint to the flight controller.

**Figure 7:**
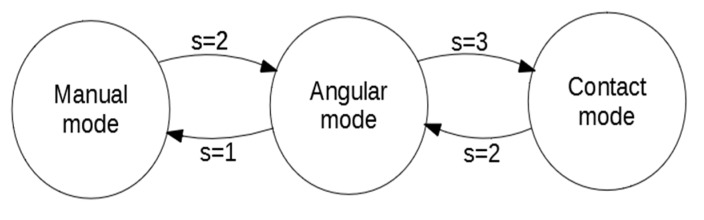
Mode switching.

The system is piloted using a radio control. This radio is an input for the payload that calculates the roll, pitch and yaw targets and sends these data to the flight controller to control the UAV. The control system has been divided into three modes (Figure 7), using a three position switch (s) of the radio control transmitter to switching between modes:

- *Manual mode*: In this mode, the radio inputs are directly transmitted to the flight controller as targets.
- *Angular mode*: In this mode, the controller of the payload calculates the Yaw target to control the angle formed between the UAV and the structure.
- *Contact mode*: In this mode, the controller of the payload calculates the Yaw as before and the Pitch target to control the approaching of the UAV to wall and the contact.

The control signals of the angular and contact modes are limited, so the UAV moves slowly and in a controlled fashion. For safety reasons, the radio signals always overwrite the control signals generated by the payload, so the operator of the UAV can always control the system regardless of the mode in which the system is. In this way, if the system is destabilized or lose control, the operator can control it manually to stabilize it.

#### 2.2.1. Angular Mode

**Figure 8:**
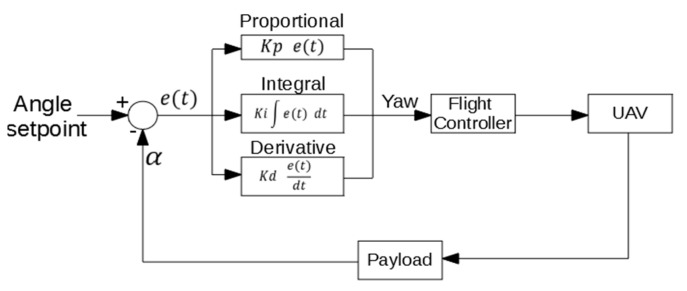
PID algorithm for the angular control.

This first mode has been designed to help the operator to place the UAV parallel to the structure before the approaching starts. In this mode, a PID algorithm actuates over the Yaw signal with the angle calculated by the payload as input. Figure 8 shows the control diagram of this mode. In this case, the objective is to be parallel to the structure, so the angle setpoint is 0°.

As aforementioned, the payload measurements are not reliable for angles near to 0°, and, hence the PID does not actuate when the measured angle is between −4° and +4°. In that case, it is considered that the UAV is completely parallel to the structure. A possible solution for this problem could be to install the distance sensors of the payload with a small angle offset, but this would make the angular range of the system shorter.

To avoid possible collisions when the system starts to rotate near to a wall, a security clause has been added. When the absolute value of the angle formed between the UAV and the structure is greater than 10°, the system only acts on the Yaw signal if the UAV is at a safe distance from the structure. This safety distance depends on the UAV framework, and for the conducted research has been set as 60 cm. As mentioned, the frame coordinate-system is centered in the joint for the distance sensors. As a result, when the UAV is in contact with the structure, the measured distance is 35 cm.

#### 2.2.2. Contact Mode

**Figure 9:**
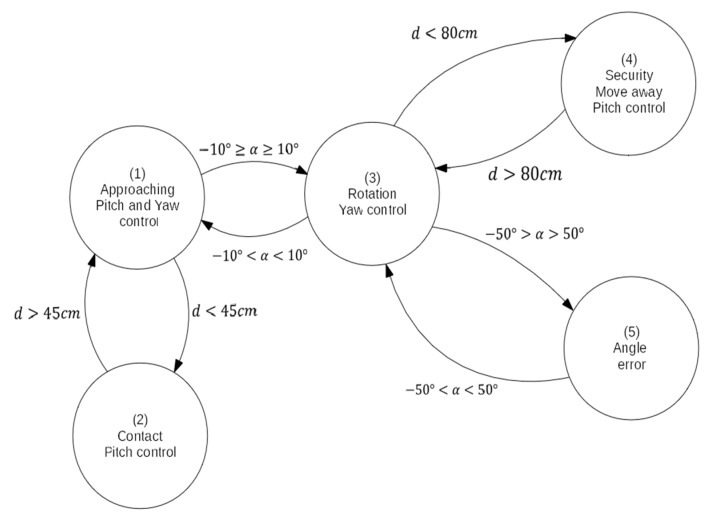
Hybrid automaton of the contact mode.

This control mode is similar to the previous one, using a PID algorithm to control the approaching to the structure. In this mode, two signals are controlled: yaw and pitch. In a first development, just one control system was designed to control pitch and yaw continuously, but better results have been obtained using a control system based on states that varies the control strategy according to the UAV status. In order to control this switching between states, a hybrid automaton has been applied (Figure 9), whose hybrid states correspond to the possible operative modes in the contact mode [30].

When the system is switched to the contact mode, it begins in the first state (approaching), in which the system actuates on the pitch and yaw signal to control the approach of the UAV to the structure. When the UAV is near the structure during the approach, the system switches to the second state and stop controlling yaw signal. If the value of the absolute angle formed between the UAV and the structure is greater than 10°, the system switches to the third state, in which it stops controlling the pitch signal and just controls the yaw one to make the UAV parallel to the structure. Once the UAV is parallel to the structure, the system switches to the first state again and continues with the approaching. If the system is not parallel to the structure and the distance is less than the minimum safe distance, it switches to the fourth state, in which it actuates on the pitch signal to move the UAV away from the structure. Once the safety distance is reached, the system switches to the third state again and begin controlling the yaw signal to make the UAV parallel to the structure. Also, a fifth state has been added. The system switches to this state if the angle formed by the UAV regarding to the structure is greater/smaller than a maximum/minimum defined angles. In this state, the system does not actuate on any signal and the operator has to manually control the UAV to move it to a valid angle, where the system switches to the second state.

**Figure 10:**
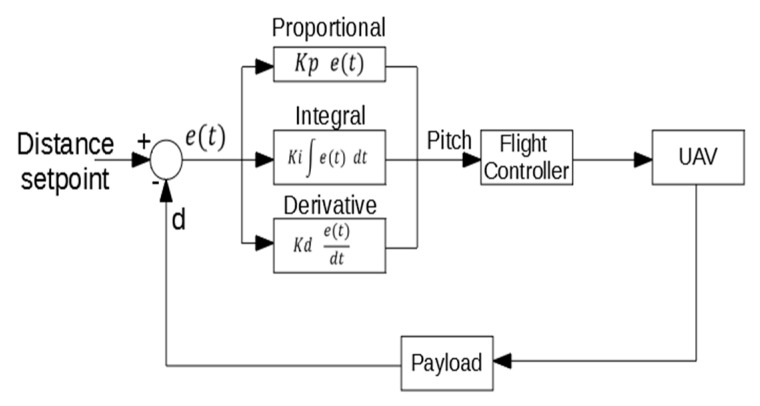
PID algorithm for the approaching control.

As stated above, five states have been defined in this hybrid automaton, each one representing a possible state of the UAV during the approaching:

1. ***Approaching*:** In this state, the controller actuates over the pitch and yaw target. A PID control algorithm similar to the PID used for the angular control has been implement for the approaching control (Figure 10). For the angle control, the same PID algorithm of the angular mode is used. For this state, the distance setpoint is set to 0. As mentioned before, when the payload is in contact with the structure the measured distance is 35 cm. This means that the UAV continue trying to move towards the structure when is already in contact, which causes the system to generate a force against the structure in a stable contact. All the mechanical components have been fabricated using a 3D printer, making them a bit flexible. This flexibility helps the system to make a stable contact.
2. ***Contact*:** In this state, the controller only actuates over the pitch signal, using the same PID algorithm for the approaching of the previous state.
3. ***Rotation*:** In this state, the controller only actuates over the yaw signal. The control is done with the same PID algorithm of the angular mode.
4. ***Security*:** In this state, the controller actuates on the pitch signal with the same PID algorithm than the approaching state, but changing the distance setpoint to the minimum safe distance. This makes the system move away from the structure.
5. ***Angle error*:** In this state, the system does not actuate on any signal. The operator has to control the UAV manually in this state.

**Figure 11:**
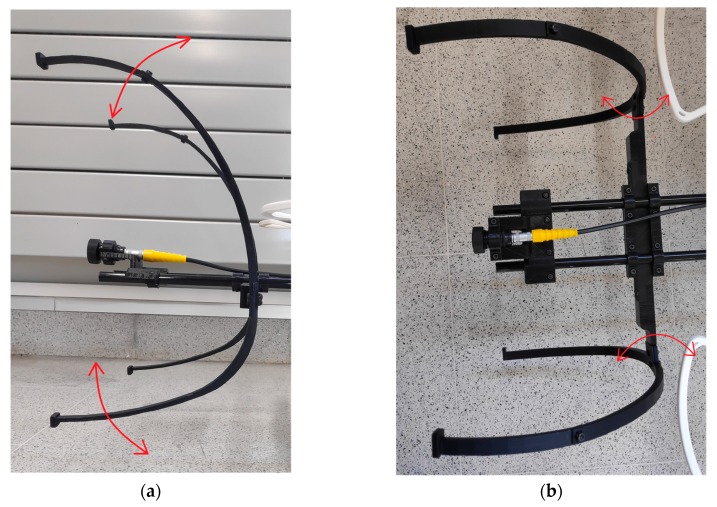
Flexible support legs for the contact with the bending movement (red arrows). (**a**) Side view. (**b**) Top view.

If the angle formed by the UAV and the structure is between −4° and +4°, the system considers that is parallel to the structure. Due to this, the system is not able to control the angle to place the contact sensor completely parallel to the structure. In order to solve this problem, four support legs for contact have been added to the designed payload. These legs have been 3D printed and have some flexibility (Figure 11).

## 3. Results and Discussion 

### 3.1. Case Study

**Figure 12:**
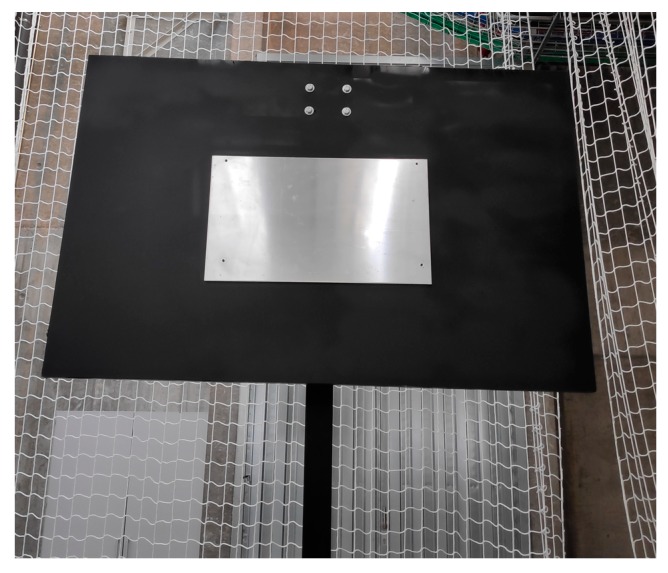
Structure used for the tests.

The developed payload has been tested in an indoor environment, in a laboratory of the MTI (Centro de Investigación Tecnolóxica Industrial) of the University of Vigo. The payload has been designed to work without GPS and any other external positioning system. It only uses the sensors on board on the UAV. To simulate a contact in an elevated metallic structure, a metal sheet has been fixed on a plastic panel two meters high (Figure 12). In this way, the tests can be performed without the ground effect.

The dimensions of the metal sheet used for the experiments are 60 cm wide and 40 cm high, with a nominal thickness of 6 mm. The metal sheet is fixed to a black plastic flat support of 1.5 m wide and 1 m high. An Arduino Due (Arduino.CC, Italy) has been used as the payload controller. For the sensor connection, a series of PCBs have been designed and fabricated. For the coupling of the payload to the UAV carbon fiber tubes have been used, achieving a rigid union without a significantly weight increasing.

### 3.2. Experimental Results 

A series of test have been done. First, the system has been tested using a simulated contact sensor fabricated with a 3D printer. These first tests have been done for tuning the gains of the PID controllers.

**Figure 13:**
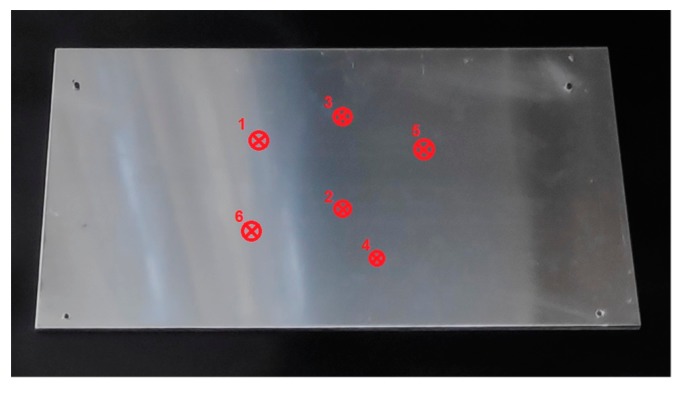
Position of the contacts done during the tests.

Once the gains of the PID have been tuned, a series of contact experiments with the structure designed for the tests have been done. In total, six contact inspections have been done in different parts of the metal sheet (Figure 13).

The thickness measured in the six experiments was always 5.4 mm. In order to check if the difference with the nominal value was due to spurious errors induced by the UAV measuring technique, manual measurements were made at different points of the metal sheet, including the points where the UAV established the contacts. In all of the points the measurements obtained were the same, 5.4 mm, the value obtained by the UAV. This difference between the nominal thickness of the metal sheet and the ultrasonic measurements could be avoided with a specific sensor calibration for small thickness ranges. This difference does not appear for ranges higher than 10 mm. In any case, it is demonstrated that it is a specific problem of the ultrasonic sensor and not of the contact measurement system based on UAV shown in this work.

The ultrasonic sensor used is especially designed to use onto UAVs. The sensor has two types of membrane for contact, one of them needs the wet gel couplant and the other does not. With the membrane that does not need the gel, called dry couplant membrane, measurements present a greater error. Due to this, authors decided to use the membrane with the gel for the tests. A dose of gel is sufficient to perform up to 4 measurements, after which the gel must be replaced in the membrane. 

**Figure 14:**
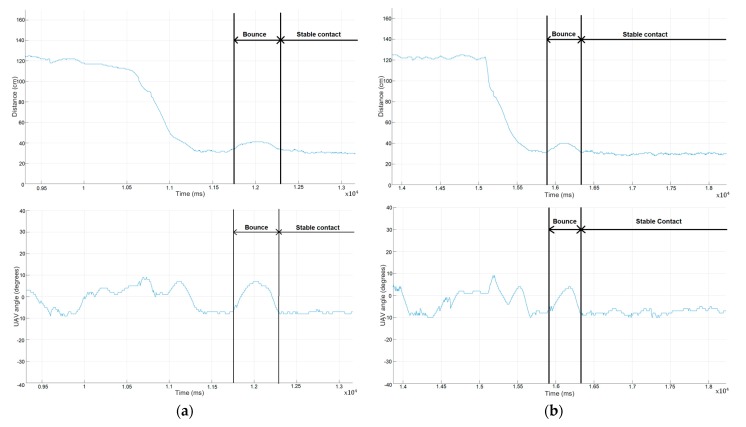
Angle and distance of the UAV regarding the structure during the approaching and the contact. (**a**) First contact. (**b**) Second contact.

As can be seen in Figure 14, when the UAV contacts the structure it bounces. After this small bounce, the system is able to maintain a stable contact with the structure. Also, during the bounce the system rotates but the flexibility of the mechanical components absorbs that rotation. 

When the UAV is in contact with the structure, the formed angle is stable but not 0°. This means that during the contact, the UAV is not parallel to the structure, but the support of the sensor is able to absorb this angle. In this way, the sensor is completely in contact with the structure, making a good measurement. During the contact, the controller does not actuate over the yaw signal.

When the UAV is approaching to the structure, some air flows created by the propellers and the proximity to the structure affects to the UAV, making it rotate on the vertical axis. The controller is able to control these rotations, keeping the angle value between −10° and 10°, which is enough for the mechanical components to fix the angle and make the measurement. 

**Figure 15:**
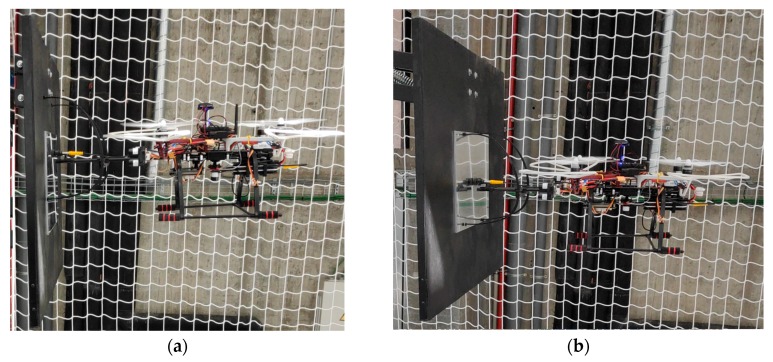
Image of the UAV during the contact. (**a**) Side view. (**b**) Oblique view.

As can be seen in Figure 15, when the UAV is in contact with the structure the UAV is slightly tilted forward, with a small pitch angle. This means that it is applying a force on the structure. The mechanical supports have been designed to be flexible, so they absorb this small angle, making a complete contact between the sensor and the structure. 

Potential improvements have been found during the design and testing of the payload. Some are simple improvements, such as increase the base line between the distance sensors to improve the resolution of the angle measurement. Other are more complex ones such as the design of a mechanical system to absorb the bounce of the system that occurs at the first contact.

## 4. Conclusions 

The advances in the design of flight control systems for UAV allow these systems to be used in different fields. The principal advantage of these systems is that they are able to reach difficult access areas easily, economically and safely:

- Nowadays, many studies have been done to control a UAV to perform a contact inspection tasks, but in most of them, positioning systems external to the UAV are used. These systems require the installation of a series of equipment (i.e. cameras, beacons) in the area where the UAV will fly, which makes this not directly applicable to a real case in which this equipment could not be installed to perform the inspection.
- The presented payload is able to perform contact inspection tasks without any other positioning systems. This payload is able to measure the distance between the UAV and the structure and the angle they form, making possible to control the approaching and the contact. Also, this payload is independent of the flight controller, making it adaptable to other frames configurations or flight controllers.
- Future trends include improving the navigation on the axis parallel to the structure and improving automation from the current system, that is able to perform semi-automatic contact inspections, to a future fully automated system.
